# The influence of team motivational climate on employee creativity—mediating role of domain-relevant skills

**DOI:** 10.3389/fpsyg.2023.1177778

**Published:** 2023-10-10

**Authors:** Yanmei Zhao, Ting Yu, Zhengtang Zhang

**Affiliations:** ^1^Department of Human Resource Management, School of Business, Nanjing Audit University, Nanjing, China; ^2^Department of Human Resource Management, Business School, Nanjing University, Nanjing, China

**Keywords:** team motivational climate, mastery climate, performance climate, domain-relevant skills, employee creativity

## Abstract

Employee creativity drives enterprise development, and team motivational climate plays an important role in incubating employee creativity. Based on creativity component theory, this study explores the impact of team motivational climate (mastery climate and performance climate) on employee creativity and its mechanism. Through the paired data of supervisors and employees at three time points, the research shows that mastery climate positively affects employees’ domain-relevant skills and domain-relevant skills positively affect employee creativity. By controlling the mediating effect of intrinsic motivation and self-efficacy, domain-relevant skills mediate the impact of mastery climate on employee creativity; performance climate and mastery climate work together on domain-relevant skills. When both are high, domain-relevant skills are highest. Performance climate moderate the mediating effect of domain-relevant skills between mastery climate and employee creativity. When performance climate is high, the mediating effect of domain-relevant skills is stronger. Suggestions for practice and future research are provided.

## 1. Introduction

Innovation is the lifeblood of enterprise development (Černe et al., 2014), and improving employee creativity is the foundation of enterprise innovation (Amabile, 1988). The importance of innovation has encouraged the development of many behaviors aimed at cultivating employee creativity, among which creating a specific team motivational climate is one important way to stimulate employee creativity. Team motivational climate includes mastery and performance climates (Ames, 1992). However, contrary to the emphasis on a competitive performance climate, some enterprises encourage harmony among employees and recommend that they learn from each other to promote high creativity. The question then arises as to what kind of motivational climate is more conducive to stimulating employee creativity: a competitive performance climate or a mastery climate that emphasizes learning and mastering knowledge. Do the two exist simultaneously and have a superimposed effect? What are the underlying mechanisms? These questions are of relevance to both business and academia.

Many studies have focused on the impact of team climate on employee creativity, such as team innovational climate (Ingram, 2016) and innovation support climate (Diliello et al., 2011; Tsai et al., 2015; Xu and Luo, 2016; Qu et al., 2019). Some studies focused on the influence of team motivational climate (including performance climates and mastery climates) on employee creativity. Mastery climates refer to the work structure in which individuals value effort, sharing, and collaboration with a focus on learning and mastering skills (Nerstad et al., 2013). On the other hand, performance climates refer to the work structure in which individuals value performance comparisons and demonstrating superiority over other employees (Nerstad et al., 2013). However, the conclusions toward the relationship of team motivational climate on employee creativity are inconsistent or even contradictory. For example, Liu (2013) found that a competitive performance climate between teams positively influenced team creativity. However, Zhu et al. (2018) found that team performance climate did not directly affect employee creativity. Liu and Chen (2017) found that team performance climate negatively affected the creativity of employees in a team. Other studies explored the moderating effect of team performance climate and team mastery climate on the relationship between external environment and employee creativity. For example, Černe et al. (2014) found that both team mastery climate and team performance climate moderated the relationship between employee knowledge hiding and employee creativity. Bari et al. (2019) also found that team mastery climate can alleviate the negative effects of knowledge hiding on team creativity.

Although many studies have focused on the impact of team motivational climate on employee creativity, there remain research gaps. From one perspective, relatively few studies have directly investigated the main effect of team motivational climate on employee creativity. Most studies only considered team motivational climate (mastery climate and performance climate) as a moderating variable (e.g., Černe et al., 2014, 2017; Bari et al., 2019). From another perspective, even studies that focused on the impact of team motivational climate on employee creativity derived inconsistent and even conflicting conclusions. Unclear results can lead researchers to draw incorrect conclusions and leave business managers at a loss for appropriate actions. Therefore, clarifying the influence of team motivational climate on employee creativity is particularly important.

To address the problem, this study clarifies the effect of team motivational climate on employee creativity. We propose that mastery climate and performance climate may synergistically affect employee creativity, as mastery climate provides employees motivation to gain knowledge, while performance climate provides employees with the direction of accumulating knowledge. To gain an in-depth understanding of the effect of team motivational climate on employee creativity, drawing upon creativity component theory (Amabile, 1988, 1996), this study reveals the mediating role of domain-related skills through which team mastery climate influences employee creativity. According to this theory, team mastery climate may stimulate employees to learn and accumulate domain-related skills, which is the foundation for employees to generate new ideas and thus improve employee creativity. A performance climate moderates the relationship between team mastery climate and domain-related skills and thus the mediation effect of domain-related skills in the link between team mastery climate and employee creativity, because it provides the impetus for employees to accumulate domain knowledge.

## 2. Theory and hypotheses

### 2.1. The influence of mastery climate on domain-relevant skills

According to achievement goal theory, team motivational climate refers to the common views of criteria for success and failure formed by employees through policies, practices, and procedures in the work environment (Nicholls, 1984; Nerstad et al., 2013). Team motivational climate includes mastery climate and performance climate. Team mastery climate supports effort and collaboration, and emphasizes learning, mastery, and the development of capabilities (Ames, 1992). In a mastery climate, employees tend to cooperate and share information (Ames and Archer, 1988), and thus build trusting relationships (Ommundsen et al., 2003). Team mastery climate supports efforts and cooperation among team members, and emphasizes learning, mastery, and the development of competencies (Ames, 1992). In a mastery climate, employees are more likely to cooperate and share information (Ames and Archer, 1988), and thus develop trusting relationships (Ommundsen et al., 2003).

Domain-relevant skills refers to the knowledge and skills accumulated by employees in a specific field, which is the basis of employees’ high creativity (Amabile, 1988). Team mastery climate can motivate employees to accumulate domain-related skills in three ways. First, mastery climate emphasizes learning, mastery, and ability development (Ames, 1992). As a motivational climate, a mastery climate motivates employees to learn and master new knowledge (Nerstad et al., 2013). In a mastery climate, employees are more likely to master and accumulate domain-relevant skills by constantly exploring and learning. Second, the process of in-depth study, learning, and accumulation of new knowledge is often accompanied by boredom, and the learning process requires willpower. In a mastery climate, employees will have higher commitment to work, make extra efforts, and be more resilient when facing difficulties (Ntoumanis and Biddle, 1999; Roberts, 2012; Nerstad et al., 2013), therefore, such employees are more persistent when they experience the mundane aspects of knowledge accumulation. Finally, employees tend to have higher performance in a mastery climate (Nerstad et al., 2013). When both employees and their colleagues have higher performance, this, in turn, will act on employees and motivate them to make greater efforts to master new knowledge. Based on the above review, it is clear that team mastery climate can improve employees’ domain-relevant skills. Thus, we proposed the following hypothesis:

*H1*: Team mastery climate is positively related to employees’ domain-relevant skills.

### 2.2. The influence of domain-relevant skills on employee creativity

According to creativity component theory, three elements are required for employees to display creativity: intrinsic motivation, innovation skills, and domain-relevant skills (Amabile, 1988). Domain-relevant skills are the knowledge background foundation of employees’ innovation, linking the influence of employee factors and environmental factors to employee creativity (Amabile, 1996). Existing studies of the impact of environment on employee creativity have paid more attention to the mediating role of intrinsic motivation (e.g., Shalley et al., 2004; Liu et al., 2016, 2018); little attention has been paid to domain-relevant skills and many such skills have been considered only at the level of theory (Liu et al., 2016). However, according to creativity component theory, both intrinsic motivation and domain-relevant skills are the core factors of the external environment that affect employee creativity (Amabile, 1988, 1996).

Creativity often occurs in a specific domain. To be creative in a domain and achieve theoretical breakthroughs, it is necessary to master the domain knowledge (Duarte Alonso et al., 2018). Mastering domain-relevant skills can not only help employees identify problems and find innovation points, but also inspire the generation of creative ideas (Liu et al., 2017). The greater the individual’s domain-relevant skills, the more alternatives that individual has available by which to innovate products and generate new ideas (Amabile 1996). In a sense, domain-relevant skills determine the cognitions of employees during the creative process (Amabile and Pillemer, 2012). Employees with a wealth of domain-relevant skills are more likely to develop a good understanding of the nature of a problem and combine and recombine different knowledge to produce creative solutions. In addition, these knowledge and skills are also resources themselves, and when available, they are likely to be used to address new challenges in the process of creativity.

Furthermore, the positive impact of domain-relevant skills on employee creativity has also been supported by empirical studies (e.g., Liu et al., 2017; Emami et al., 2023). Although Liu et al. (2017) did not make direct assumptions regarding domain-relevant skills and employee creativity, the research results showed that domain-relevant skills can mediate the influence of high-performance human resource systems on employee creativity. Both the derivation and the results showed that domain-relevant skills positively affect employee creativity. Their study is the first empirical study of domain-relevant skills, which lays a foundation for exploring the impact of domain-relevant skills on employee creativity. Additionally, Emami et al. (2023) found that, when controlling for intrinsic motivation and creativity-relevant processes, domain-relevant skills are positively associated with the creativity of upcoming engineers. Based on the above review, it appears that domain-relevant skills can improve employee creativity. Thus, we proposed the following hypothesis:

*H2*: Employees’ domain-relevant skills are positively related to employee creativity.

### 2.3. The mediating role of domain-relevant skills

According to achievement goal theory (Nicholls, 1984), mastery climate as a type of team motivational climate that emphasizes learning and mastering knowledge as the criterion of success will motivate employees to keep learning and mastering new knowledge. When encountering difficulties in the learning process, employees will have higher resilience given a mastery climate (Ntoumanis and Biddle, 1999; Roberts, 2012; Nerstad et al., 2013), and subsequently develop skills within the field. Creativity component theory posits that domain-relevant skills are the basis of employee creativity. As a knowledge resource, a high level of domain-relevant skills can help employees identify innovation points and find innovation breakthroughs: such skills can help employees generate alternative solutions to solve innovation problems (Liu et al., 2017). Having a knowledge base makes it easier for employees to adopt and integrate the ideas of others, which, in turn, is also beneficial for employee creativity (Grant and Berry, 2011; Zhao et al., 2023). However, employees with limited field-relevant skills often lack basic knowledge pertaining to that field. Even given a team motivational climate, they tend to miss innovation opportunities. In the face of innovation problems, a lack of knowledge will hinder the generation of innovative ideas. Based on the above analysis, it is clear that a mastery climate can improve employee creativity through domain-relevant skills. The following hypothesis was therefore proposed:

*H3*: Domain-relevant skills mediate the influence of mastery climate on employee creativity.

### 2.4. The moderating role of performance climate

In contrast to a mastery climate, another dimension of the team motivational climate is performance climate, which emphasizes the criteria of success (Nicholls, 1989; Roberts, 2012). In a performance climate, teams emphasize group standards, social comparisons, and intra-team competition (Ames and Ames, 1984). Only those who win such competitions are considered successful (Ames, 1984). In a performance climate, in which individual performance is often compared to that of others, such as by ability grouping and verbal comparison, a competitive relationship may develop among employees because their goal is to perform better than their colleagues (Ames and Ames, 1984).

A mastery climate stimulates employees to learn and master new domain knowledge, whereas a performance climate provides the impetus for employees to accumulate domain knowledge. In a mastery climate, the team emphasizes learning and mastering new knowledge, but the motivation may be directionless; that is, employees are more likely to explore independently in a mastery climate, which is helpful for employees to accumulate knowledge, but the knowledge accumulated may not directly improve their work. By contrast, a performance climate influences employees’ understanding and perception of what is valued and expected (Halisah et al., 2021), which provides the direction for employees to learn and accumulate domain-relevant knowledge. In a team that has both a mastery climate and a performance climate, both knowledge and performance are used to define employee success (Nicholls, 1989; Roberts, 2012). In addition to continuing to learn and master knowledge, employees also need to achieve high performance to obtain the recognition of their team. In synergy, the motivation to acquire knowledge is more likely to be directed toward domain-relevant skills because it implies higher performance. Therefore, a combined performance climate and mastery climate will synergistically affect employees’ domain-relevant skills, and creativity related skills should be highest when both climates are present.

Although no research to date has tested the synergistic effect of performance climate and mastery climate on employees’ domain-relevant skills, Liu et al. (2017) found that supportive human resource and performance human resource systems can synergistically affect employees’ domain-relevant skills. A supportive human resource system that seeks growth and stability and a performance human resource system that seeks high performance can effectively promote the accumulation of domain-relevant skills of employees. Buch et al. (2015) also found that a mastery climate is positively associated with individual intrinsic motivation, and a performance climate moderates this relationship. Based on the above analysis, it is likely that a performance climate can regulate the relationship between mastery climate and employees’ domain-relevant skills. The following hypothesis was therefore proposed:

*H4*: Team performance climate and team mastery climate interact with employees’ domain-relevant skills. When both are high, employees’ domain-relevant skills are the highest.

Similarly, a mastery climate encourages employees to learn and explore knowledge, and employees in this climate are more resilient and persistent in the face of challenges in the learning process (Ntoumanis and Biddle, 1999; Roberts, 2012; Nerstad et al., 2013). Continuous learning and exploration help employees build skills relevant to their domain. As a resource for creativity, the accumulated knowledge in their domain lays a foundation for employees to find innovation points and generate creative solutions, which help employees to fully express high creativity.

While a mastery climate provides motivation for employees to learn, a performance climate provides direction for employees to learn. In a team with both a mastery climate and a performance climate, employees’ learning is more likely to be directed toward achieving high performance, and they are more likely to accumulate domain-relevant skills that contribute to such performance, thus promoting employee creativity. However, in a low performance climate, although driven by a mastery climate, employees maintain the motivation to learn, but the learning direction does not necessarily relate directly to field-relevant knowledge, as employes may explore content outside their field based on their own interests. As a result, the accumulation of domain-relevant skills is likely relatively limited and the resulting creativity will be diminished. Based on the above analysis, team performance climate likely regulates the mediating effect of domain-relevant skills. The following hypothesis was therefore proposed:

*H5*: Performance climate moderates the mediating effect of domain-relevant skills on the relationship between performance climate and employee creativity. When performance climate is high, the mediating effect of domain-relevant skills is stronger.

**Figure 1:**
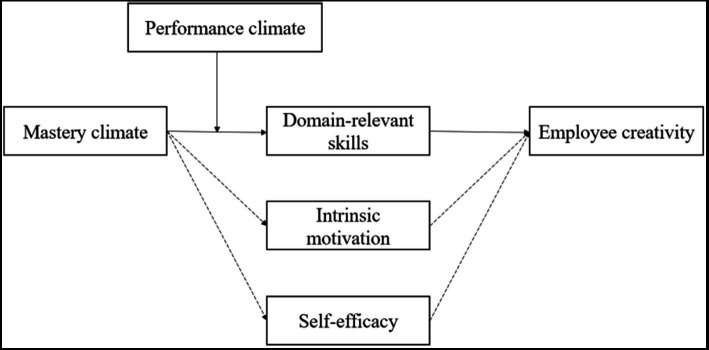
Theoretical model.

Although a mastery climate may motivate employees to apply domain-relevant skills, in practice, in high-tech enterprises that emphasize innovation, both climates influence the accumulation of employees’ domain-relevant skills rather than their application. According to creativity component theory, the relationship between domain-relevant skills and employee creativity is direct and not amenable to adjustment by other factors. Therefore, this study does not consider the moderating effect hypothesis regarding the second stage (between domain-relevant skills and employee creativity). The model of this study is shown in Figure 1.

## 3. Method

### 3.1. Sample and procedures

Our study involved two companies, which are a network technology company and a robot education & training company. Both of which place a high value on employee creativity, making them appropriate samples for this study. Prior to conducting the survey, the researchers contacted the general managers of the two companies and obtained the companies’ roster. Employees on the roster were invited to participate in our survey. In the introduction, they were informed orally and in writing that their participation was voluntary and that they could quit anytime. Subsequently, the researchers conducted several interviews with each company’s human resource department and employees, and made appropriately adjustments to enhance their situational applicability.

To minimize homologous variance and social desirability, the survey employing a three-time-point data collection method for employee-leader pairings. This method follows the interval duration of one week used by Lin et al. (2017) in previous studies on creativity. Specifically, time 1 involved employee evaluations of mastery climate, performance climate, job creativity requirement and organizational emphasis on creativity, as well as the demographic variables such as age, gender, post duration and industry duration. Time 2 consisted of domain-relevant skills, intrinsic motivation and self-efficacy. Finally, time 3 involved direct supervisors evaluating employees’ creativity.

The survey collected 301 employee questionnaires at time 1, and 265 employee questionnaires at time 2. Additionally, the creativity evaluation of 258 employees by 47 supervisors were collected at time 3. A total of 234 valid samples were successfully matched through the 3 times of survey. Among them, 156 and 78 samples were collected from the robot training and network technology companies, respectively. The sample comprised 42.7% women, 72.2% under 30 years old, and 64.1% had an undergraduate degree or above.

### 3.2. Measures

To ensure congruence of the Chinese version with the English version of the scales, we followed the widely used translation and back-translation method (Brislin, 1986).

#### 3.2.1. Mastery climate

Mastery climate was measured with a six-item scale developed by Nerstad et al. (2013). Employees rated items such as “In my team, one is encouraged to cooperate and exchange thoughts and ideas mutually.” Likert 5 scoring method was adopted (1 = very strongly disagree and 5 = very strongly disagree). This scale has also been applied in the study of the Černe et al. (2014). In this study, the Klonbach coefficient *α* = 0.92.

#### 3.2.2. Performance climate

Performance climate was measured with eight-item scale developed by Nerstad et al. (2013). Employees rated items such as “In my team, it is important to achieve better than others.” Likert 5 scoring method was adopted (1 = very strongly disagree and 5 = very strongly disagree). In this study, the Klonbach coefficient *α* = 0.88.

#### 3.2.3. Domain-relevant skills

Domain-relevant skills was measured using five-item scale developed by Youndt et al. (2004) and later adapted by Liu et al. (2017). Employees rated items such as “I am highly skilled in my job/specialty area” (*α* = 0.89). Likert 7 scoring method was adopted (1 = very strongly disagree and 7 = very strongly disagree).

#### 3.2.4. Employee creativity

Employee creativity was measured using four items (Hirst et al., 2011). Items were “Generates ideas revolutionary to the field,”“Seeks new ideas and ways to solve problems,” “Is a good role model for innovation/creativity,” and “Tries new ideas and approaches to problems.” Team leaders rated employees’ creativity on a scale ranging from 1, “very strongly disagree,” to 7, “very strongly agree”(*α* = 0.83).

#### 3.2.5. Control variables

In previous studies on creativity, age, education level, post duration, industry duration, job creativity requirement and organizational emphasis on creativity may affect employee creativity (Farmer et al., 2003; Casimir et al., 2012; Gong et al., 2013; Lin et al., 2017), so we controlled for them in this study. Referring to the treatment of education level in Jia et al. (2014), this study treated education level and age as continuous variables. Job creativity requirements was measured using scales developed by Casimir et al. (2012) and organizational emphasis on creativity was measured by scale developed by Farmer et al. (2003). The Klonbath coefficients are 0.86 and 0.92, respectively. Finally, according to the theory of creativity components, intrinsic motivation is also the key factor that external environment affects employee creativity (Amabile, 1988), and Liu et al. (2016) found that employee self-efficacy is also one of the three driving factors that external environment and personal factors affect employee creativity. Therefore, this study controls the mediating effects of intrinsic motivation and self-efficacy when discussing the mediating effects of domain-relevant skills.

## 4. Results

### 4.1. Confirmatory factor analysis

To verify the discriminative validity of the variables, confirmatory factor analysis was performed. The factor analysis results are shown in Table 1 for mastery climate, performance climate, domain-relevant skills, employee creativity, job creativity requirements, organizational creativity emphasis, intrinsic motivation, and self-efficacy. Table 1 shows that an eight-factor model with eight independent variables fit the data well (*χ*^2^ (296) = 564.72, CFI = 0.95, TLI = 0.94, RMSEA = 0.06, SRMR = 0.05); all indexes met their criteria for good fit, and the model listed was significantly better than other alternative models. This analysis indicates that there was a good discriminative validity among the variables.

**Table 1:** Confirmatory factor analysis results.

	*χ* ^2^	df	*χ*^2^/df	CFI	TLI	RMSEA	SRMR
8 factors	564.72	296	1.91	0.95	0.94	0.06	0.05
7 factors	1116.48	303	3.68	0.83	0.81	0.11	0.09
6 factors	1276.08	309	4.13	0.74	0.70	0.13	0.11
5 factors	1871.84	314	5.96	0.68	0.65	0.15	0.12
4 factors	2025.01	318	6.37	0.65	0.62	0.15	0.12
3 factors	2381.94	321	7.42	0.58	0.54	0.17	0.13
2 factors	2639.73	323	8.17	0.53	0.49	0.18	0.13
1 factor	3110.69	324	9.60	0.43	0.39	0.19	0.14

8 factors = job creativity requirement, organizational creativity support, mastery climate, performance climate, domain-relevant skills, intrinsic motivation, self-efficacy, employee creativity.

### 4.2. Descriptive statistics

Table 2 displays the means, standard deviations, correlations, and reliability values of all variables. As can be seen from Table 2, domain-relevant skills were significantly correlated with age (*r* = 0.17, *p* < 0.01), job duration (*r* = 0.15, *p* < 0.05), and industry duration (*r* = 0.28, *p* < 0.01), indicating that analyses should adjust for these variables. There was a significant correlation between mastery climate and employees’ domain-relevant skills (*r* = 0.31, *p* < 0.01), and domain-relevant skills were significantly correlated with employee creativity (*r* = 0.24, *p* < 0.01); this provides a foundation for exploring the mediating effect of domain-relevant skills on the relationship between mastery climate and employee creativity.

**Table 2:** Descriptive statistics and correlations.

	Mean	SD	1	2	3	4	5	6	7	8	9	10	11
1.Age	2.91	0.95											
2.Education	3.58	0.72	−0.26										
3.Job tennure	2.76	1.44	0.44**	−0.12									
4.Industry tenure	3.25	1.43	0.52**	−0.12	0.70**								
5.Creativity requirement	3.82	0.72	0.04	−0.03	0.10	0.08							
6.Organizational creativity support	3.91	0.69	0.04	−0.04	0.001	0.06	0.64**						
7.Mastery climate	4.00	0.58	0.05	−0.01	0.03	0.05	0.38**	0.41**					
8.Performance climate	3.52	0.62	0.05	−0.02	0.14*	0.13*	0.38**	0.37**	0.37**				
9.Domain-relevant skills	4.67	0.95	0.17**	−0.03	0.15*	0.28**	0.36**	0.32**	0.31**	0.43**			
10.Self-efficacy	5.5	0.85	0.15*	−0.05	0.03	0.11	0.38**	0.43**	0.58**	0.42**	0.57**		
11.Intrinsic motivation	3.89	0.73	0.12	−0.12	−0.03	−0.02	0.41**	0.43**	0.57**	0.32**	0.36**	0.59**	
12.Employee creativity	3.28	0.58	0.03	0.01	0.04	0.16*	0.06	0.12	0.14*	0.05	0.24**	0.15*	0.04

*p < 0.05, **p < 0.01, ***p < 0.001.

### 4.3. Hypotheses testing

SPSS 24.0 was used for regression analysis, and Mplus 7.11 was used to build the moderated mediation model to test the main research hypotheses of this study. Table 3 displays the regression analysis results. Models 1–3 (M1–M3) used domain-relevant skills as the dependent variable, whereas models 4–7 (M4–M5) used employee creativity as the dependent variable.

**Table 3:** Results of hierarchical regression analyses.

Variables	Domain-relevant skills			Employee creativity	
	M1	M2	M3	M4	M5
Age	0.05	0.05	0.05	−0.03	−0.04
Education	0.02	0.02			
Job tennure	−0.08	−0.08	−0.09	−0.05	−0.04
Industry tenure	0.2***	0.2***	0.18***	0.11**	0.08*
Creativity requirement	0.34***	0.29**	0.22*	−0.03	−0.07
Organizational creativity support	0.18	0.11	0.05	0.11	0.09
Mastery climate		0.28**	0.2*		
Performance climate			0.33***		
Mastery climate * Performance climate			0.41**		
Domain-relevant skills					0.12**
R^2^	0.21	0.23	0.33	0.48	0.80
⊿R^2^	0.21	0.02	0.10	0.48	0.32
*F*	9.95***	9.77***	11.93***	1.90	2.79**

*p < 0.05, **p < 0.01, ***p < 0.001.

#### 4.3.1. Main effect

To test the influence of mastery climate on employees’ domain-relevant skills, employees’ domain-relevant skills were used as the dependent variable in a regression on mastery climate. According to Model 2, after adjusting for age, education level, job duration, industry duration, job creativity requirement, and organizational emphasis on creativity, the influence of mastery climate on employees’ domain-relevant skills was significant (*β* = 0.28, *p* < 0.01), which supported hypothesis 1.

To test the influence of domain-relevant skills on employees’ creativity, employee creativity was used as the dependent variable in a regression on domain-relevant skills. According to Model 5, after adjusting for age, education level, job creativity requirement, and organizational emphasis on creativity, the influence of domain-relevant skills on employee creativity was significant (*β* = 0.12, *p* < 0.01), which supported hypothesis 2.

#### 4.3.2. Mediating effect

Bootstrapping and path analysis were used to test the mediating role of domain-relevant skills, as suggested by Liu et al. (2012). According to creativity component theory, intrinsic motivation is the key factor by which the external environment affects employee creativity (Amabile, 1988). Further, Liu et al. (2016) found that employee self-efficacy is one of three external environment and personal factors that drive employee creativity. Therefore, the mediating roles of intrinsic motivation and self-efficacy were controlled in this study in the path analysis that tested the mediating role of domain-relevant skills. When adjusting for the mediating effects of intrinsic motivation and self-efficacy, the path analysis showed that domain-relevant skills significantly mediated the relationship between mastery climate and employee creativity (*β* = 0.03, *p* = 0.05), which initially supported hypothesis 3. Bootstrapping with 2000 replications indicated a 99% confidence interval of the mediating effect of domain-relevant skills on mastery climate and employee creativity that excluded zero [0.002, 0.101]. Therefore, the mediating effect was significant, which supported hypothesis 3.

#### 4.3.3. Moderating effect

To test the moderating effect of performance climate on mastery climate and employees’ domain-relevant skills, SPSS24.0 software was used to centrally process the data of performance climate and mastery climate. On this basis, two interaction terms entered the regression based on employees’ domain-relevant skills (see Table 3, Model 3). After adjusting for control variables and main effects, the interaction terms significantly influenced domain-relevant skills (*β* = 0.41, *p* < 0.01); that is, the influence of performance climate on employees’ domain-relevant skills was moderated by mastery climate, which initially supported hypothesis 4.

**Figure 2:**
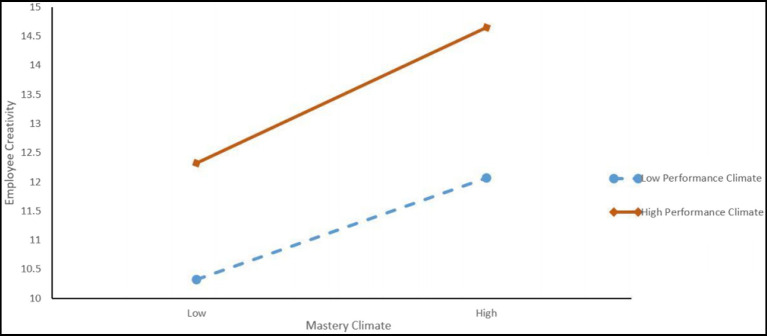
Interaction of mastery climate predicting employee creativity.

On the basis of the significant moderating effect, to further explain the strength and direction of the moderating effect of performance climate, values one standard deviation above and below the mean of the moderating variable were used to generate a plot, as suggested by Baron and Kenny (1986) (Figure 2). As can be seen from Figure 2, for employees in a high performance climate, mastery climate had a stronger effect on employees’ domain-relevant skills, which supported hypothesis 4.

#### 4.3.4. Moderated mediation effect

Bootstrapping was adopted to test the moderated mediation effect. The results of bootstrapping with 2000 replications is shown in Table 4. As can be seen from Table 4, the 95% confidence interval of the moderated mediating effect excluded zero [0.010, 0.174], indicating that employee performance climate significantly moderated the mediating effect of employee domain-relevant skills on the relationship between mastery climate and employee creativity.

**Table 4:** Results of moderated mediation.

Moderator	Indirect effect	Standard error	95%CI
High performance climate	0.057	0.034	99% [0.010, 0.147]
Low performance climate	−0.007	0.018	95% [−0.060, 0.018]
Difference	0.064	0.039	95% [0.010, 0.174]

Specifically, when employees perceived a high performance climate, their domain-relevant skills significantly mediated the relationship between mastery climate and employee creativity; the 99% confidence interval excluded zero [0.010, 0.147]. However, when employees perceived a low performance climate, the mediating effect of employees’ domain-relevant skills on the relationship between mastery climate and employee creativity was not significant; the 95% confidence interval included zero [−0.060, 0.018]. This shows that only in the high-performance climate did employees’ domain-relevant skills mediate the influence of mastery climate on employee creativity.

## 5. Discussion and conclusions

Based on creativity component theory, this study explored the additive effect of mastery climate and performance climate on employee creativity and the mediating role of domain-relevant skills. Through a paired-data study of supervisors and employees at three time points, mastery climate was shown to have a significant positive impact on domain-relevant skills, and a high mastery climate motivated employees to master domain-relevant knowledge. Domain-relevant skills can mediate the influence of mastery climate on employee creativity; performance climate and mastery climate have a synergistic effect. In a high performance climate, a mastery climate has a stronger influence on employees’ domain-relevant skills, and the mediating effect is stronger on the relationship between mastery climate and employee creativity.

### 5.1. Theoretical contribution

First, the study illuminates the impact of team motivational climate on employee creativity. This study found that as one of the dimensions of team motivational climate, mastery climate can positively affect employee creativity through domain-relevant skills. Although the influence of team motivational climate on employee creativity has been considered in prior research, such extant work has not reached consensus. For example, Liu (2013) found that an inter-team competitive (performance-oriented) climate had a positive effect on team creativity, whereas Zhu et al. (2018) found that an inter-team competitive performance atmosphere did not directly affect employee creativity. However, Liu and Chen (2017) found that a performance-motivated climate negatively affects the creativity of employees. Although these studies focused on the influence of a performance climate within a motivational climate on employee creativity, they seem to indicate that the influence of motivational climate on employee creativity is unstable. The inconsistency of research conclusions suggests that more studies are needed to clarify the relationship between the aforementioned variables. The current empirical study found that another dimension of team motivational climate, namely mastery climate, can positively affect employee creativity by improving their domain-relevant skills. This adds to the existing literature on the impact of motivational climate on employee creativity and helps to clarify prior discrepancies in research results regarding these two types of climate. Future research can explore the impact of the two types of team climate on other aspects of employees, such as positive work behavior and organizational citizenship behavior.

Second, this study found an additive effect of mastery climate and performance climate, thereby extending the existing literature. Although many studies have explored the impact of motivational climate on employee creativity, almost all examined only one dimension of motivational climate, such as the impact of performance climate on employee creativity (e.g., Liu, 2013; Liu and Chen, 2017; Zhu et al., 2018). However, studies that concurrently considered performance climate and mastery climate only adopted these climatic variables as moderators of the influence of environmental factors on individual creativity, ignoring the possibility of a superimposed effect of performance climate and mastery climate on employee creativity; that is, creativity may be greatest when both forms of climate are present. This study found that mastery climate and performance climate interact in their effect on employee creativity through domain-relevant skills, and verified that the two have a superimposed effect on employee creativity. This is an important supplement to existing research on the influence of motivational climate on creativity. Based on this finding, future research can explore other potential boundary conditions that determine how team motivational climate affects employee creativity.

Third, this study directly examined the mediating role of domain-relevant skills, which is an important addition to the literature on creativity components. The results showed that domain-relevant skills mediated the influence of motivational climate on employee creativity. Many previous studies have focused on the impact of environmental and individual factors on employee creativity (Shalley et al., 2004; Liu et al., 2016, 2017; Vincent and Kouchaki, 2016), but most studies use internal motivation as a mediating variable (Shalley et al., 2004; Liu et al., 2016). According to creativity component theory, employees’ domain-relevant skills are also an indispensable factor in the influence of the external environment on employee creativity. Based on the control of intrinsic motivation and self-efficacy, this study examined the mediating role of domain-relevant skills on the relationship between mastery-oriented climate and employee creativity, which represents direct verification of creativity component theory and an important supplement to the theoretical literature. Future research can explore other possible mechanisms by which a motivational climate affects employee creativity, drawing on other theories. For instance, researchers can investigate the role of self-efficacy based on social cognitive theory.

### 5.2. Managerial implications

First, enterprises should create a mastery climate to stimulate employee creativity. Team mastery climate will affect employee creativity through their domain-relevant skills. Team mastery climate is the basis for motivating employees to learn and accumulate knowledge and skills in the field, and thereby achieve high creativity. In the future, enterprises should attach importance to creating a mastery atmosphere within organizations and teams, and encourage employees to learn and master new knowledge. For example, organizations should be constructed as learning organizations, emphasizing the concept of continuous and lifelong learning, to stimulate the learning motivation of employees and promote high creativity.

Second, enterprises should create a performance climate to stimulate employee creativity. Team mastery climate and performance climate synergistically affect domain-relevant skills, and thus affect employee creativity. When both climates are highly present, employees’ domain-relevant skills are higher and the mediating effect is stronger. In the future, enterprises should not cultivate only one kind of climate among teams. The mastery and performance climate have additive effects, and hence promoting each of these climates should receive equal attention. For example, enterprises should encourage employees to learn and master new skills while measuring their success or failure according to their performance. The concurrent cultivation of performance and mastery climates is mutually complementary, jointly promoting employee creativity, and realizing the unity of short-term performance and long-term development of the enterprise.

Third, enterprises should focus on improving employees’ field-relevant skills. Employees’ domain-relevant skills can mediate the influence of team motivational climate on employee creativity and play a key role in the process of stimulating employee creativity. In the process of cultivating employee creativity in the future, enterprises should not only pay attention to the intrinsic motivation considered in previous studies, but also to cultivating employees’ domain-relevant skills. For example, the enterprise might provide training for employees and invite experts in the field to provide professional guidance for employees. Only when domain-relevant skills are improved can employees give full expression to creativity in their work.

### 5.3. Limitations and directions for future research

This study found that domain-relevant skills can mediate the influence of motivational climate on employee creativity, and at the same time control the mediating effects of intrinsic motivation and self-efficacy. However, according to the theory of creativity components, employee creativity depends on intrinsic motivation, domain-relevant skills, and creativity skills (Amabile, 1988). Although domain-relevant skills and intrinsic motivation among the three factors were tested in this study, there was no direct verification of creativity skills. Future research is necessary to develop a scale based on the theory to examine the mediating role of creativity skills in the relationship between external environment and employee creativity.

Considering that the climate perceived by employees has the most direct impact on employee behavior, this study adopted team performance climate and mastery climate as individual perceptions and relegates them to the individual level for testing. Although this approach has been supported by other studies, such as Liu et al. (2017), which tested two organizational level variables (organizational performance human resource system and security human resource system) at the individual level, this approach also meant that the current study lacked team-level variables. In the future, the hypothesis of this study should be tested again, this time at the team level.

## Data availability statement

The raw data supporting the conclusions of this article will be made available by the authors, without undue reservation.

## Author contributions

TY and YZ performed the material preparation, data collection, and analysis. TY wrote the first draft of the manuscript. ZZ commented on previous versions of the manuscript. All authors contributed to the study conception and design, read, and approved the final manuscript.
